# B-Raf and the inhibitors: from bench to bedside

**DOI:** 10.1186/1756-8722-6-30

**Published:** 2013-04-25

**Authors:** Tiangui Huang, Michael Karsy, Jian Zhuge, Minghao Zhong, Delong Liu

**Affiliations:** 1 Department of Pathology, Westchester Medical Center and New York Medical College, Valhalla, NY 10595, USA; 2 Department of Neurosurgery and Department of Urology, Westchester Medical Center and New York Medical College, Valhalla, NY 10595, USA; 3 Division of Hematology and Oncology, Westchester Medical Center and New York Medical College, Valhalla, NY 10595, USA

## Abstract

The B-Raf protein is a key signaling molecule in the mitogen activated protein kinase (MAPK) signaling pathway and has been implicated in the pathogenesis of a variety of cancers. An important V600E mutation has been identified and can cause constitutive B-Raf activation. Recent studies have evaluated a variety of small molecule inhibitors targeting B-Raf, including PLX4032/vemurafenib, dabrafenib, LGX818, GDC0879, XL281, ARQ736, PLX3603 (RO5212054), and RAF265. Therapeutic resistance has been identified and various mechanisms described. This review also discussed the current understanding of B-Raf signaling mechanism, methods of mutation detection, treatment strategies as well as potential methods of overcoming therapeutic resistance.

## Discovery of Raf

**Figure 1:**
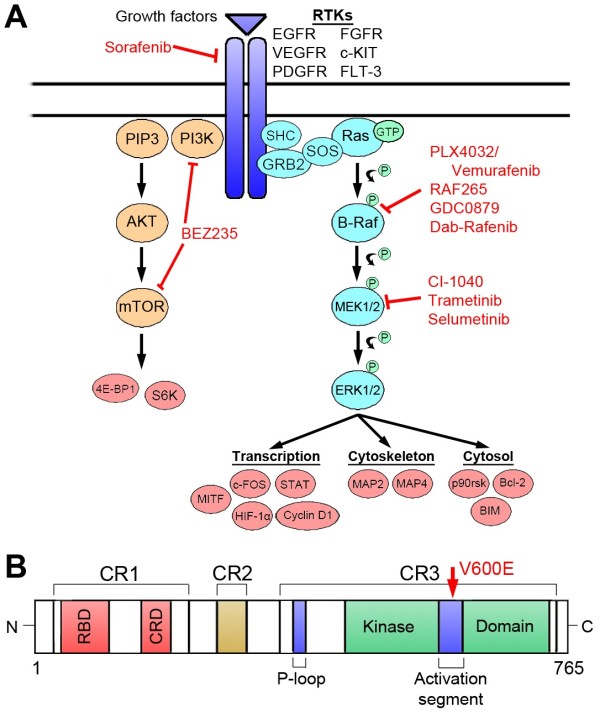
**B-Raf protein and signaling pathways.** The B-Raf protein and its related signaling pathway are shown along with potential targets for treatment. **A**) The PI3K/AKT/mTOR and Ras/Raf/MAPK signaling pathways are shown along with potential targets. **B**) The structural domains of the B-Raf isoforms are shown. The position of the V600E mutation is indicated (arrow).

The first *Raf* gene (*v-Raf*), characterized from murine sarcoma virus 3611 in 1983 by Mark and Rapp, was named after the discovery of its role in fibrosarcoma in newborn MSF/N mice [1]. Two years after its discovery, the first functional human homolog of the *v-Raf* gene, specifically *C-Raf-1*, was cloned from human cells. The *C-Raf-1* gene, also known as *Raf-1*, is composed of 80,626 base pairs of DNA with 17 exons on chromosome 3p25, which encodes a 3,291 nucleotide mRNA and 648 amino-acid peptide molecule (http://www.genecards.org). Subsequent to the discovery of *C-Raf-1*, the *A-Raf* and *B-Raf* isoforms were characterized [2,3]. The *A-Raf* gene (*ARAF1*), also termed *PKS* or *PKS2*, is located on X chromosome p11.4-11.2 and encodes a 1821 nucleotide mRNA and 606 amino-acid peptide molecule (http://www.genecards.org). Human *B-Raf*, located on chromosome 7q34, is composed of 18 exons and encodes a 2,949 bp length mRNA and 766 amino-acid residue peptide. Classically known as a serine/threonine protein kinase, *B-Raf* has also been classified as proto-oncogene B-Raf for murine sarcoma viral (v-Raf) oncogene homolog B1, and B-Raf proto-oncogene serine/threonine-protein kinase (p94). An inactive pseudogene (B-RAFP1, 3,356 bp, Gene ID: 286494) is located on chromosome Xq13 [4]. A-Raf, B-Raf and C-Raf belong to a protein-serine/threonine kinase family that along with their downstream molecules, MEK and ERK, constitute the classic mitogen activated protein kinase (MAPK) signaling pathway [5]. Each Raf isoform shares three conserved domains (Figure 1), including the N-terminus domain CR1, containing Ras-binding and cystine-rich domains; CR2, which is serine/threonine rich and contains a 14-3-3 binding site; and CR3, which is a conserved C-terminus domain that acts as a protein kinase and has a stimulatory 14-3-3 binding site [2]. There is 76% homology between the amino acid sequences of B-Raf and C-Raf, and 74% similarity between B-Raf and A-Raf [6].

Wild-type Raf functions by forming a homodimer or heterodimer with A-, B- and C-Raf isoforms (for more detail, refer to [2]). These dimers can up-regulate MEK1 or MEK2 which further act on ERK1 or ERK2, respectively. The diverse dimer patterns and their downstream diverse molecules make the Raf signal pathway very sophisticated. The Raf/MEK/ERK kinase signal pathway is highly involved in cell proliferation, differentiation and tumorigenesis [2]. Raf, including B-Raf, can regulate multiple downstream molecules and is also regulated by a variety of signaling molecules. Multiple transcription/signaling molecules such as p53, AP-1, NF-KappaB, C/EBPalpha, STAT3, c-Jun, have specific binding sites in the B-Raf promoter and may regulate B-Raf expression [7-9]. The B-Raf related PI3K/AKT/mTOR and Ras/Raf/MAPK signaling pathways and potential targets for treatment, as well as the structural domains of the B-Raf isoform are summarized in the Figure 1.

## Raf mutations in tumors

While mutations of *A-Raf* and *C-Raf* are generally rare in neoplasia, mutations of *B-Raf* have been detected in a variety of cancers. B-Raf gene mutation has been detected in approximately 45% of papillary thyroid carcinoma (PTC) [10], 50-80% of melanoma [11], ~100% of hairy cell leukemia, 11% of colorectal cancer and 41% of hepatocellular carcinoma [12-15]. Solid tumor masses can contain heterogeneous concentrations of stromal /non-neoplastic cells in comparison to leukemia, and may dilute the percentage of cells with mutant B-Raf [10]. It is important to note that a single *Raf* mutation without Ras activation provides an ideal candidate for targeted therapy since mutant Raf signals as a monomer [16]. However, if one monomer of the homodimer/heterodimer in a normal Raf protein is bound to the Raf inhibitor, the other monomer in the dimer can still be transactivated and continue to stimulate its downstream signaling pathway. Thus a sole B-Raf inhibitor will not work in this situation. For the B-Raf V600E mutation, Raf inhibitor binds to the sole Raf monomer and blocks its signal transduction.

Even though over 70 different B-Raf mutations have been detected, the V600E (T1799A) mutation in exon 15 is predominant in a variety of tumors [17]. Due to three extra nucleotides found in GC rich exon 1 of B-Raf DNA, the original V599E was changed to the V600E [17]. V600E mutation in the kinase domain results in constitutive Ras-independent activation of B-Raf, thereby facilitating signal transduction within the downstream MAPK kinase pathway and promoting cancer development [18,19]. *B-Raf* mutations involving V600E accounts for 68% and 80% of the mutation events in metastatic and primary melanoma, respectively [20].

Despite the importance of B-Raf in carcinogenesis, the role of this protein as a driver mutation remains controversial. A study conducted in 65 different melanotic lesions at different stages including nevi, radial growth phase (RGP), vertical growth phase (VGP) melanomas and melanoma metastases, revealed that *B-Raf* mutation was detected in only 10% of early stage or RGP melanoma. This suggests that *B-Raf* mutations correlated with progression rather than initiation of human melanoma [21]. Later, in a conditional *B-Raf V600E* mutation mouse model, it was shown that the expression of mutated B-Raf induced the formation of benign melanocytic hyperplasia [22]. However, these hyperplasia did not evolve into melanoma over 15-20 months. In the same study, concomitant PTEN silencing along with *B-Raf* mutation caused rapid melanoma development and metastasis. These findings suggest that *B-Raf* mutations could occur early in the progression of melanoma yet it is unclear exactly what effect these mutations have on this disease.

*B-Raf* gene mutations are also commonly detected in thyroid carcinoma. The first study of B-Raf V600E in papillary thyroid carcinoma (PTC) detected such mutation in 24/35 (69%) of cancers [23]. Additionally, there is substantial evidence showing V600E mutation in classic PTC, but not with follicular thyroid carcinoma and medullary thyroid carcinoma [24-26]. Later studies showed that the mutation rate was about 45%. Further studies showed that this mutation had a strong relationship with poor prognosis including recurrence, nodal or distant metastasis and upgraded staging as well as shortened long term survival in PTC [26,27]. Similar to melanoma, the role of V600E as an early driver mutation or later event in PTC remains controversial [28]. In one study, *B-Raf* mutation was detected in lymph nodes with metastatic PTC while the primary PTC was negative for the mutation, which suggested the mutation is a late event [29]. On the contrary, in animal models of PTC, the expression of mutant B-Raf V600E alone could initiate the development of PTC [30,31]. A very recent study found that this *B-Raf* mutation prevalence was 56.9% in 72 PTC cases, and more importantly, the B-Raf V600E allele was detected in 5.1% to 44.7% of samples, suggesting again that *B-Raf* mutation was a late event [32]. In the same study, it was shown that *N-*, or *K-Ras* mutation could co-exist with B-Raf V600E mutation in very small percentage of cases. The authors did not exclude the possibility that *Ras* or *Raf* mutation came from different cells. This observation might also explain why resistance to B-Raf inhibitors develops, since wild type *B-Raf* containing cells are resistant to B-Raf inhibitors. Further study by the same group showed that the higher the percentage of mutant B-Raf allele in the tumor, the worse the long-term survival of the patient with this mutation [33].

*B-Raf* mutation was recently reported in hairy cell leukemia. Tiacci *et al.* detected *B-Raf V600E* mutation in all hairy cell leukemia samples studied (n = 47) with whole-exome sequencing of CD19 positive leukemic cells [34]. None of 195 patients with other peripheral B cell lymphoma carried the same mutation. Further study with leukemic cells from 5 patients showed that Raf inhibitor RLX4720 significantly lowered levels of phosphorylated MEK and ERK, indicating that in hairy cell leukemia, mutated B-Raf was most likely the trigger for constitutive MEK and ERK activation. Similar findings from another study by a pyrosequencing assay showed that B-Raf V600E mutation in classic hairy cell leukemia was seen in 79% of samples [35]. The investigators excluded the cases which had hairy cell leukemia in <10% of total peripheral blood cells, therefore the sensitivity could not explain the difference of these two studies.

## Detection methods of *B-Raf* mutations

Various methods have been investigated for the detection of *B-Raf* mutations, with more recent techniques showing greater sensitivity and throughput [10]. Sanger sequencing is traditionally a reliable method and considered the “gold standard” technique for mutation detection [36]. This technique has low throughout and requires several distinct steps, namely PCR amplification with dideoxynucleotide end termination, amplicon purification, and sequence reading. Each of these steps exposes samples to risk of contamination, and has low analytical sensitivity of 10-20% [36,37]. A newer technique including pyrosequencing, the technique used for the next generation of sequencing, was found to be superior to direct sequencing in detecting B-Raf mutations when a low abundance of mutant templates were present [38]. However, pyrosequencing requires expensive equipment thereby making it impractical for most laboratories. Nonetheless, the advantage of sequencing is that other variants beyond V600E can also be detected in a single run.

Two other traditional methods for B-Raf mutation detection include the PCR-restriction fragment length polymorphism (PCR-RFLP) and PCR single-strand conformation polymorphism (PCR-SSCP) assays. PCR-RFLP utilizes sequence amplification and enzymatic digestion followed by comparison to known DNA sequence samples. PCR-SSCP involves sequence amplification and sample electrophoresis in order to exploit base pair differences resulting in secondary cDNA structure alterations. These two are labor-intensive and require considerable personnel experience to employ. PCR-RFLP has a detection sensitivity of 1-3% for detecting mutations of B-Raf V600E [39,40], while the PCR-SSCP, by incorporating with Sanger sequencing, has a sensitivity of 5% [15].

Newer methods are emerging in the detection of gene mutations in clinical samples. Shifted termination assay (STA) was developed by the TrimGen Corporation involving rapid analysis of a 96-well microplate using a fluororimetric detection [41]. The three mutations in the *BRAF* gene V600E, A, or G can be accurately detected by both fluorescent color and fragment size. The sensitivity is 1–5%.

Allele-specific PCR is another category of mutation detection methods with the advantage of mutant enrichment and high sensitivity. The goals of these methods are to increase sensitivity, specificity and to accurately quantify mutations of B-Raf V600E. One type of allele-specific PCR is the amplification refractory mutation system (ARMS)-PCR, a highly sensitive, specific and low-cost method for detecting B-Raf mutations. Using this technique, our laboratory recently developed an ARMS-PCR test for V600E with a sensitivity of 0.5% [10]. In a single tube, four primers are added to generate three products with one serving as control and favoring mutant product forming by adjusting more mutant specific primer. With the advance of real-time technique, Lang *et al*., developed an ARMS real-time PCR method with a sensitivity of ~1% [42]. Morandi *et al*. developed an allele-specific locked nucleic acid (LNA) quantitative PCR assay using LNA-modified allele specific primers and LNA-modified beacon probes [36]. Furthermore, the detection sensitivity was 0.1% and the entire procedure can be completed in 3 hours. This method was successfully used for monitoring circulating B-Raf mutation in melanoma patients after biochemotherapy [43].

A few commercial kits are available to detect B-Raf mutations. Dual-priming oligonucleotide (DPO)-based multiplex PCR [37] is used in Seeplex B-Raf auto-capillary electrophoresis detection system. The DPO system has two separated primer segments with the 5′ segment being longer than the 3′ segment. The two joined by a non-annealing poly(I) linker. The longer 5′ segment preferentially binds to the template DNA and initiates stable annealing, whereas the shorter 3′ segment selectively binds to its target site and can specifically target V600E mutations, thereby avoiding nonspecific annealing. The declared sensitivity is 2% [44]. Furthermore, Qiagen distributed an assay for B-Raf V600E, Ecomplex, D, K or R mutation using a Rotor-Gene Q (RGQ)-PCR kit with real-time PCR on the RGQ instrument (Qiagen BRAF RGQ PCR Handbook). Using ARMS and scorpions technologies, this method has a sensitivity of 1.27%. The Cobas® 4800 BRAF V600 mutation test from Roche can detect V600E, D or K at sensitivity of 5% with real-time PCR.

## B-Raf inhibitors in early phase development

Recent developments in targeting B-Raf are changing the treatment of various oncological diseases. A first generation multi-target kinase inhibitor, sorafenib (Nexavar, or BAY 43-9006), was initially developed as a Raf inhibitor and tested for melanoma [45,46]. However, this agent failed to yield a significant improvement in survival as a single drug. Further investigation revealed that sorafenib also targeted VEGFR, PDGFR, Flt-3, c-kit and FGFR-1 [47]. Recently, sorafenib was approved for the treatment of renal cell carcinoma and hepatocellular carcinoma mainly because of anti-angiogenesis effects rather than Raf inhibition [5,48,49].

**Figure 2:**
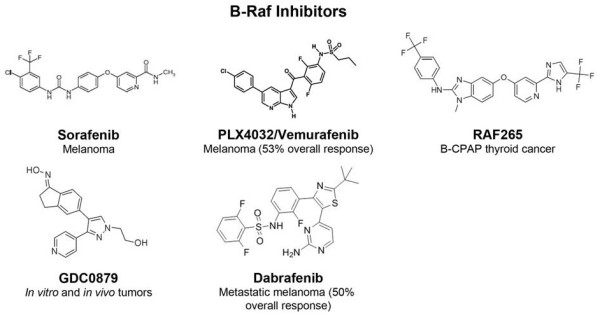
B-raf inhibitors in clinical development.

The second generation of Raf inhibitors has improved selectivity and efficacy (Figure 2). PLX4032’s activity against B-Raf V600E mutation yielded intense excitement for melanoma therapy. PLX4032 binds the ATP-binding domain of mutant B-Raf monomer and, under the condition that Ras is not constitutively activated, can block MEK and ERK signaling [50,51]. Potent anti-cancer effects are balanced against side-effects including skin rash, and the development of squamous cell carcinoma in about 30% of patients [5].

Several novel small molecules targeting mutant B-Raf are being actively evaluated in preclinical models and early clinical trials (Table 1; clinicaltrials.gov). GDC0879 was tested in 130 tumor cell lines and tumor bearing mice [52]. The results revealed that the inhibitory function was associated strictly with the B-Raf V600E status of the cells. The treatment improved the survival rate of mice harboring the B-Raf mutant tumor MEXF989 compared to the mutant K-Ras–expressing tumor MEXF535.

**Table 1:** B-Raf inhibitors in phase I/II clinical trials

**Drugs**	**Disease**	Clinical trial ID*
LGX818	Advanced melanoma	NCT01436656
Dabrafenib (GSK2118436)	Solid tumors	NCT00880321
XL281	Solid tumors	NCT00451880
ARQ-736	Advanced solid tumors	NCT01225536
RO5212054 (PLX3603)	Advanced solid tumors	NCT01143753
RAF265	Advanced melanoma	NCT00304525

*details available at http://www.clinicaltrials.gov.

LGX818 is a potent and selective RAF kinase inhibitor with little activity against wild-type BRAF. In vitro studies showed no significant activity against a panel of 100 kinases (IC_50_ > 900 nM). LGX818 did not suppress the growth of > 400 cell lines expressing wild-type BRAF. LGX818 treatment at oral doses as low as 6 mg/kg in human melanoma xenograft models (BRAF^V600E^) resulted in strong and sustained (>24 hours) decrease in pMEK [53]. A Phase I clinical trial in patients with BRAF mutant tumors is ongoing (NCT01436656).

XL281 is a selective inhibitor of RAF kinase with anti-tumor activity in xenograft models. A phase I clinical study of XL281 orally once daily on a 28-day cycle in patients with colorectal (CRC), melanoma, papillary thyroid (PTC) and NSCLC [54]. The dose escalation phase enrolled 30 pts. DLTs included fatigue, nausea, vomiting, and diarrhea at the dose 225 mg. The MTD was 150 mg. The most common treatment-related AEs included Grade 1/2, fatigue (48%), diarrhea (35%), nausea (35%), vomiting (35%) and anorexia (30%). Severe AEs included hypokalemia, nausea, and vomiting. Biomarker studies from paired biopsies from 4 pts (3 melanoma, 1 NSCLC) showed decreases in pMEK, pERK, and Ki67. MTD of XL281 was established at 150 mg.

ARQ736 is an ATP-competitive pan-RAF kinase inhibitor. This small molecule is a potent inhibitor of mutant BRAF (V600E), wild-type BRAF and c-RAF, in colon, melanoma and thyroid cancer cell lines as well as in xenograft mouse models [55,56]. A phase I clinical study has been initiated.

PLX3603 (RO5212054) is also being tested in a phase I clinical trial in patients with advanced solid tumors (NCT01143753) [57].

RAF265 represents a novel oral small molecule dual inhibitor of mutant BRAF^V600E^ (EC_50_ = 0.14 μM) and VEGFR2 (EC_50_ = 0.19 μM). RAF265 was demonstrated in B-CPAP thyroid cancer cells to abrogate downstream ERK signaling [58]. Furthermore, this study showed that RAF265 also inhibited RET activity. When combined with phosphoinositol-3-kinase (PI3K)/ mammalian target of Rapamycin (mTOR) inhibitor BEZ235, a synergistic effect leading to decreased cell proliferation was observed. A first-in-human dose escalation study was done in 76 patients to identify MTD, PK, PD and anti-tumor activity of RAF265 [59]. Different schedules were tested: daily (QD), weekly and intermittent. 8 dose levels were administered. Six of the 71 evaluable pts reported DLTs within 1^st^ cycle (28 days): pulmonary embolism (2), visual disturbances (2), hyperlipasemia (1), diarrhea (1) and ataxia (1). Grade III and IV thrombocytopenia was found to be also dose limiting. In conclusion, the MTD of oral RAF265 was determined to be 48 mg daily. An intermittent schedule at 67 mg higher dose is being explored.

## B-Raf inhibitors in phase III studies

In 2011, the competitive small molecule serine/threonine kinase inhibitor, vemurafenib (Zelboraf, PLX4032), was approved by FDA for the treatment of patients with unresectable or metastatic melanoma harboring the B-Raf V600E mutation [60]. Vemurafenib was tested in a phase III clinical trial in 132 metastatic melanoma patients (Table 2). Among them, 122 patients were harboring B-Raf V600E and 10 harboring V600K mutation. The dose was 960 mg orally twice daily until the development of unacceptable toxic effects or disease progression. The overall response rate was 53% in a 12.9-month follow-up period (range 0.6 to 20.1). The median duration of response was 6.7 months, and median progression-free survival was 6.8 months. Importantly, only 14% of patients had disease progression. The common side effects of vemurafenib included grade 1 or 2 arthralgia, rash, photosensitivity, fatigue and alopecia. Unfortunately, 26% of patients developed cutaneous squamous-cell carcinoma in the trial.

**Table 2:** B-Raf inhibitors in phase III clinical trials

**Drugs/regimen**	**Diseases**	**Dosage**	Clinical trial ID*
Dabrafenib	Advanced melanoma	Dabrafenib	NCT01682083
(GSK2118436)		150 mg BID	NCT01584648
+Trametinib (GSK1120212)		±Trametinib 2 mg OD for 12 months	NCT01597908
Vemurafenib (PLX4032)	Advanced melanoma	Vemurafenib 960 mg BID	NCT01597908
+ Dacarbazine		±Dacarbazine 1000 mg/m2 IV Q3weeks for 12 months	NCT01667419 NCT01689519
+ GDC-0973		± GDC-0973 60 mg QD for 21 of 28 days	NCT01006980
Sorafenib	Renal cell carcinoma	Sorafenib 400 mg BID	NCT00478114
+multiple combinations	Hepatocellular carcinoma	+multiple combinations	NCT01135056
	Acute myeloid leukemia		NCT01371981
	Stage III/IV melanoma		NCT00111007
	Non-small cell lung cancer		NCT00449033
	Pancreatic cancer		NCT00541021

*details available at http://www.clinicaltrials.gov.

The reversible B-Raf inhibitor, dabrafenib (GSK2118436), which selectively inhibits B-Raf V600E mutant, was recently investigated in clinical trials [61-63]. Dabrafenib was given at 150 mg twice daily in the phase II part of a phase I/II study [64]. Cutaneous squamous carcinoma was observed in 11% of the patients in the study. The authors concluded that dabrafenib was safe in patients with solid tumors, and was an active inhibitor of V600-mutant BRAF with responses noted in patients with melanoma, brain metastases, and other solid tumors.

The phase III study of dabrafenib was a randomized controlled trial among 250 patients with metastatic melanoma harboring B-Raf V600E [61]. In the study, 187 patients received dabrafenib and 63 (37 male and 26 female) patients received dacarbazine for comparison. The dose was oral dabrafenib 150 mg twice-daily or intravenous dacarbazine 1000 mg/m^2^ every 3 weeks. Dabrafenib -treated group reached 5.1 month of median progression-free survival versus 2.7 months with dacarbazine. Dabrafenib achieved a 50% response rate while the dacarbazine control group only achieved 6%. Treatment-related side effect occurred in 53% and 44% of patients in dabrafenib and dacarbazine groups, respectively. This study also found 12 patients (6%) developed keratoacanthoma /squamous-cell carcinoma of the skin in the dabrafenib group.

Recent studies have evaluated tumor samples after treatment with these novel B-Raf inhibitors. A study of 21 tumor samples, 11 of them treated with vemurafenib, found that keratoacanthoma or cutaneous squamous-cell carcinoma developed as early as 3 weeks after vemurafenib treatment, with a mean incubation time of 10 weeks [65]. The *Ras* mutation was detected in 13 samples with 12 containing *H-Ras* mutation. Further biochemical analysis showed that tumor specimen had higher ERK phosphorylation than surrounding normal skin. Vemurafenib was also used to treat murine cell line B9 harboring *H-Ras* mutation and found to paradoxically increase MAPK signaling and cell proliferation. The effect of B-Raf inhibitor on carcinoma formation was further confirmed by the spontaneous tumor formation in animal treated by DMBA-TPA, a known *H-Ras* mutation inducer with or without PLX4720, a B-Raf inhibitor [65]. DMBA-TPA and PLX4720 combination reduced the latency of the squamous cell carcinoma formation and PLX4720 paradoxically increased MAPK signal in the cell lines harboring mutated *H-Ras*[65].

Multiple mechanisms have been suggested to support the clinical efficacy of B-Raf inhibitors. Activation of the Ras/Raf/MAPK pathway plays an important role in cancer development. Theoretically, interference of this pathway might achieve an anti-tumor effect, which is at least, partially true in cell culture, xenograft tumor models, and clinical specimens [18,66]. Raf and MEK inhibitors can decrease this signaling pathway, and therefore lead to clinic effects [50,67,68]. In another clinical study of 37 specimens from 15 patients, a raf inhibitor significantly increased CD4 and CD8 lymphocyte infiltration in the tumor. The increase of CD8 lymphocytes was correlated with reduction of tumor volume and increase of necrosis [69].

## Mechanisms of resistance to B-Raf inhibitors

Raf inhibitors have shown clinical efficacy in B-Raf V600E containing melanoma. Resistance to Raf inhibitors has been a widely explored topic. Multiple mechanisms of resistance are being uncovered [18,70].

Interestingly, Raf inhibitors suppress the ERK signaling in mutant B-Raf cells but enhance ERK signaling in wild-type B-Raf cells. B-Raf inhibitors bind to one member of the C-Raf/B-Raf heterodimer or C-Raf/C-Raf homodimer. While inhibiting one protomer (partner), this allows transactivation of the other drug-free protomer [50]. It is generally accepted that the Ras and *B-Raf* mutation is mutually exclusive. Therefore in *B-Raf* mutated tumors, Ras is not activated. Thus, transactivation of wild-type Raf with Ras is minimal. It is hypothesized that Ras activity levels in B-Raf V600E tumors may not be high enough to support the transactivation of ERK signal [50]. In one study, Raf inhibitors did not inhibit ERK signaling in cells co-expressing B-Raf V600E and mutant Ras [50]. These results suggest that an increase in Ras activation or Raf dimerization may be sufficient to cause drug resistance. Because of heterogenicity of tumors, detection of cells with mutant B-Raf does not exclude the possibility that the other tumor cells could harbor wild-type B-Raf or mutant Ras. The coexistance of different clones can lead to resistance to B-Raf inhibitors. This has been confirmed by a recent study which showed that B-Raf V600E allele only accounted for 5.1-44.7% of B-Raf alleles in the papillary thyroid carcinoma cells [32].

Altered parallel signaling has also been shown to mediate resistance to B-Raf inhibitors. In one study, chronic treatment with B-Raf inhibitor of the cell lines induced resistance as the tumor cells with B-Raf mutation can rewire their signaling properties by using any of the three active Raf isoforms, i.e. A-Raf, B-Raf and C-Raf, to trigger ERK activation [71]. In this study, inhibition of one or two isoforms did not terminate ERK activity. The resistant lines, however, showed enhanced IGF-IR/PI3K signaling. Another report showed that in cells resistant to vemurafenib, the levels of Ras-GTP, EKR and C-Raf as well as phosphorylated AKT increased [18]. Interestingly, this study demonstrated that a combination of vemurafenib with a MEK inhibitor showed a synergistic inhibitory effect on the growth of both cells and tumor xenograft. These constitute proof-of-principle that IGF-1R/PI3K/AKT mediated signaling is associated with B-Raf inhibitor resistance.

In addition, distinct mechanisms of MAPK activation have been seen during B-Raf inhibitor treatment. Mutually exclusive PDGFR beta receptor tyrosine kinase (RTK)-mediated activation and Ras-mediated reactivation of the MAPK pathway were also found to account for resistance to B-Raf inhibitors [72]. This study utilized PLX4032-resistant melanoma cell lines and further validated findings in clinical tumors and tumor matched cultures. Moreover, by expressing 597 sequence-validated kinase open reading frame (ORF) in A375 melanoma cell lines harboring B-Raf mutation, treatment with the MAPK agonist PLX4723 activated ERK primarily through a MEK-dependent mechanism without Raf signaling, and therefore facilitated *de novo* Raf inhibitor resistance [73].

Another mechanism of resistance to vemurafenib relates to V600E alternative splicing. In one study, splicing out exon 4-8 but retaining RAS binding domain generated a new p61B-Raf (V600E) isoform, which had enhanced dimerization in cells with low levels of Ras activation as compared to full-length of B-Raf (V600E) [74]. This study also showed that endogenous expression or ectopic induction of p61B-Raf (V600E) induced ERK signaling resistant to vemurafenib, where disruption of p61B-Raf (V600E) dimerization by site-directed mutation restored its sensitivity to vemurafenib. More importantly, these authors identified B-Raf (V600E) splicing variants lacking the Ras-binding domain in 6 out of 19 patients with acquired resistance to vemurafenib. This study further confirmed in clinical specimens that alternative splicing is one of the mechanisms of tumor resistance.

## Possible strategies to conquer resistance

Several strategies are being employed to overcome the resistance to B-Raf inhibitors. The combination of B-Raf inhibitor with a MEK inhibitor was found to have a synergistic or additive effect in both cell lines and xenograft models [18]. Inactivation of IGF-IR/PI3K pathway, by simultaneous MEK and IGF-1R/PI3K inhibition, has also been shown to induce cell death in B-Raf resistant cells [71]. It has been generally accepted that B-Raf and Ras mutations tend to be mutual exclusive. Simultaneous mutations of Ras and B-Raf have been reported [33]. These dual mutations along with Ras mutant/B-Raf wild type cells pose resistance to Raf inhibitor and necessitate different treatment strategies. Inhibiting combinations of B-Raf and other key signaling molecules such as Ras, or AKT may provide another way to overcome therapeutic resistance [67]. Combination with radiotherapy and general chemotherapy are also possible solutions.

## Conclusion and future directions

Significant progress has been made in the development of Raf inhibitors, detection of common mutations, and understanding the role of this key signaling molecule in carcinogenesis. Novel inhibitors of downstream molecules are undergoing active clinical trials [75].

## Competing interests

The authors have no relevant conflicts of interest.

## Authors’ contributions

All authors have contributed to data preparation, drafting and revising the manuscripts. All authors have read and approved the final manuscript.
